# Th1-Dominant CD4^+^ T Cells Orchestrate Endogenous Systematic Antitumor Immune Memory After Cryo-Thermal Therapy

**DOI:** 10.3389/fimmu.2022.944115

**Published:** 2022-07-08

**Authors:** Peng Peng, Yue Lou, Junjun Wang, Shicheng Wang, Ping Liu, Lisa X. Xu

**Affiliations:** School of Biomedical Engineering and Med-X Research Institute, Shanghai Jiao Tong University, Shanghai, China

**Keywords:** tumor ablation, cryo-thermal therapy, antitumor immunity, CD4^+^ T cells, Th1, IFN-γ

## Abstract

Recent studies suggest that highly activated, polyfunctional CD4^+^ T cells are incredibly effective in strengthening and sustaining overall host antitumor immunity, promoting tumor-specific CD4^+^ T-cell responses and effectively enhancing antitumor immunity by immunotherapy. Previously, we developed a novel cryo-thermal therapy for local tumor ablation and achieved long-term survival rates in several tumor models. It was discovered that cryo-thermal therapy remodeled the tumor microenvironment and induced an antigen-specific CD4^+^ T-cell response, which mediated stronger antitumor immunity *in vivo*. In this study, the phenotype of bulk T cells in spleen was analyzed by flow cytometry after cryo-thermal therapy and both CD4^+^ Th1 and CD8^+^ CTL were activated. In addition, by using T-cell depletion, isolation, and adoptive T-cell therapy, it was found that cryo-thermal therapy induced Th1-dominant CD4^+^ T cells that directly inhibited the growth of tumor cells, promoted the maturation of MDSCs *via* CD4^+^ T-cell-derived IFN-γ and enhanced the cytotoxic effector function of NK cells and CD8^+^ T cells, and promoted the maturation of APCs *via* cell-cell contact and CD4^+^ T-cell-derived IFN-γ. Considering the multiple roles of cryo-thermal-induced Th1-dominant CD4^+^ T cells in augmenting antitumor immune memory, we suggest that local cryo-thermal therapy is an attractive thermo-immunotherapy strategy to harness host antitumor immunity and has great potential for clinical application.

## Introduction

Conventional tumor therapies, such as surgery, chemotherapy and radiotherapy, can still result in poor prognosis and cause serious side effects, such as chemoresistance and rapid metastasis. Immunotherapy has been rapidly developed as a promising therapy for patients with advanced cancer. The main goals of anticancer immunotherapy include the induction of effective tumor-specific immunity for the eradication of tumors and the achievement of long-term tumor-free survival. As the most precise “killer” of tumor cells, genetically engineered T-cell receptor (TCR) or chimeric antigen receptor (CAR) T cells for adoptive cell therapies is an emerging immunotherapy that redirects T cells to specifically target cancer (1). However, tumor antigen heterogeneity and tumor microenvironment remain major challenges limiting their efficacy against solid tumors.

In general, CD8^+^ T cells are considered cytotoxic T-cell subsets and CD4^+^ T cells play a crucial role in the efficient induction of CD8^+^ CTL responses. Recent studies have shown that CD4^+^ T cells are important for anticancer responses and their significance has been increasingly emphasized (2). CD4^+^ T cells may drive cancer into senescence and are capable of mobilizing both the innate and adaptive immune systems (3). Moreover, CD4^+^ T cells can promote type 1 polarization of dendritic cells (DCs) and macrophages and counter immunosuppression induced by regulatory T cells (Tregs) and myeloid-derived suppressor cells (MDSCs) (4–8). Preclinical studies show that CD4^+^ T cells mediate the crosstalk between CAR-T cells and the endogenous immune system, which is necessary for optimal CAR-T-cell efficacy to prevent tumor escape and improve long-term survival outcomes (9–11). Thus, harnessing endogenous CD4^+^ T cells to modulate the immune activity of the host immune system and activate the antitumor immune response is a promising strategy for the long-term control of cancer.

We developed a novel tumor cryo-thermal therapy through the alternative cooling and heating of tumor tissue in animal models (12). The long-term survival rate following the therapy has been observed in B16F10 melanoma, 4T1 breast cancer and CT26 colorectal cancer (13–15). A pilot study revealed that the therapy induced the functional maturation of dendritic cells, promoted CD4^+^ T cell-mediated antitumor responses, and decreased Treg cells, contributing to better therapeutic efficacy in colorectal cancer liver metastasis (CRCLM) patients (16). It was found that the innate immune system was remodeled to promote adaptive T-cell immunity (15, 17–21). More importantly, cryo-thermal-induced CD4^+^ T cells, especially neoantigen-specific CD4^+^ T cells, mediated stronger systematic antitumor immunity in the long-term (13, 14). However, the mechanism by which CD4^+^ T cells that are induced by cryo-thermal therapy mediate systematic antitumor immune memory remains unclear.

In this study, we further investigated the characteristics of CD4^+^ T cells after cryo-thermal therapy and determined their role in antitumor immune memory by tumor rechallenge and T-cell depletion. Both *in vivo* and *in vitro* experiments were performed to study the differentiation and function of other immune cells modulated by CD4^+^ T cells, and Th1 dominant over other CD4^+^ subsets to execute multiple antitumor immunologic activities in substantial reduction of accumulated MDSCs and Tregs for immunosuppression reversal. Results laid the foundation for future development of a thermo-immunotherapy strategy delivered by minimally invasive cryo-thermal therapy, to modulate the host immunological environment and prevent tumor relapse and metastases.

## Materials and Methods

### Cell Culture

B16F10 mouse melanoma tumour cell line was donated by Professor Weihai Yin at Med-X Research Institute, Shanghai Jiao Tong University. The murine mammary carcinoma 4T1 cell line was provided by Shanghai First People’s Hospital, China. B16F10 cells and 4T1 cells were cultured in Dulbecco’s Modified Eagle’s Medium (DMEM; GE Healthcare, Logan, UT) supplemented with 10% fetal bovine serum (FBS, Gemini Bio-Products, West Sacramento, CA), 100 units/mL penicillin and 100 µg/mL streptomycin at 37°C in a humidified 5% CO_2_ incubator.

### Animal Models

The female C57BL/6 and BALB/c were obtained from the Shanghai Slaccas Experimental Animal Co., Ltd. (China) and used for experimental study at the age of 6–8 weeks. Mice were housed in isolated cages and a 12 h light/dark cycle environment, feeding with sterile food and water with pH value kept at 7.5–7.8. All animal experiments were approved by the Animal Welfare Committee of Shanghai Jiao Tong University, and experimental methods were performed in accordance with the guidelines of Shanghai Jiao Tong University Animal Care (approved by Shanghai Jiao Tong University Scientific Ethics Committee). To prepare the tumour-bearing mice, approximately 5×10^5^ B16F10 tumor cells or 4×10^5^ 4T1 tumor cells were injected subcutaneously into the right flank of C57BL/6 or BALB/c mouse respectively.

### The Cryo-Thermal Therapy Procedures

The system developed in our laboratory was composed of liquid nitrogen for cooling and radiofrequency (RF) for heating. To reduce the effect of contact thermal resistance and obtain a continuous thermal delivery during the treatment, a probe was designed with a cylinder-shaped tip of 1mm in diameter for the thermal therapy of subcutaneous tumor. Twelve days after B16F10 tumor inoculation or sixteen days after 4T1 tumor inoculation, when the tumor volume reached about 0.25 cm^3^, the mice were divided randomly into two groups: tumor-bearing group without the treatment (control) and the cryo-thermal group with freezing followed by RF heating on primary tumor as previously described (13). The mice were anesthetised with intraperitoneal injection (i.p.) of 1.6% pentobarbital sodium (0.5 ml/100 g, Sigma-Aldrich, St. Louis, MO, USA). The tumor site was sanitised with 75% alcohol before the treatment. All the procedures were performed aseptically.

### Tumor Rechallenge

Study of tumor rechallenge with B16F10 cells was performed in survivors 14 days after cryo-thermal therapy. Mice were intravenously infused with 1 × 10^5^ B16F10 tumor cells, and lung tumor nodules were enumerated 18 days later. One day before and four days after tumor cell infused, monoclonal antibodies were injected to deplete target cells.

### Preparation of Single-Cell Suspension of Spleen and Flow Cytometry Analysis

Mice were sacrificed after the cryo-thermal therapy, and the spleens were collected (n=4 per group). Single-cell suspension of splenocytes was prepared using GentleMACS dissociator (Miltenyi Biotec, Bergisch Gladbach, Germany) and then treated with erythrocyte-lysing reagent containing 0.15 M NH_4_Cl, 1.0 M KHCO_3,_ and 0.1 mM Na_2_EDTA to remove the red blood cells. The cells were dispersed using 70μm mesh screens and used for flow cytometry.

For cell surface staining, the cells were stained with fluorescence conjugated antibodies at room temperature for 20 min. For intracellular cytokine staining, cells were cultured in the presence of cell activation cocktail with Brefeldin A (BFA) for 4 hours. Cells were then stained with antibodies of cell surface antigen, fixed, permeabilised and incubated with antibodies of intracellular cytokines. Transcription factors staining were conducted by True-Nuclear Transcription Factor Buffer Set (Biolegend). Data was acquired using BD FACS Aria II cytometer (BD Biosciences) and analysed using FlowJo V10 software (FlowJo LLC, Ashland, OR). Fixation Buffer, Intracellular Staining Permeabilization Wash Buffer and cell activation cocktail with BFA were purchased from Biolegend (San Diego, CA). Fluorochrome-conjugated monoclonal antibodies were purchased from Biolegend, Thermo Fisher Scientific and BD Bioscience. Zombie Violet Fixable Viability Kit and Zombie Aqua™ Fixable Viability Kit were purchased from Biolegend to assess live vs. dead status of cells. Antibodies using in this article are shown in supplementary information as key resource table.

### Depletion of T Cells *In Vivo*

For T cell depletion, the treated mice (n=6 mice per group) were injected with 250μg anti-CD4 or anti-CD8 monoclonal antibody (Biolegend), respectively. Mice were injected i.p. with 250μg monoclonal antibody (mAb), on day -1 and 4 after tumor rechallenge. The effect of mAb depletion was confirmed *in vivo* previously (13).

### Tumor Cell Growth Inhibition

For tumor cell growth inhibition assay, tumor cells were seeded into a 96-well plate at 5,000 cells/well, and cocultured with CD4^+^ T cells at 8:1 of E:T ratio. Twenty-four hours later, the immune suspension cells were removed, and the viability of tumor cells was detected by Cell Counting Kit-8 (CCK-8 Kit). For CCK-8 assay, 10 μL of CCK-8 was added into each well of culturing cells, and after 1 h of incubation, the absorbance was measured at 450 nm using the microplate reader. Background reading of medium was used to normalise the result.

### Cell Isolation of CD4^+^ T Cells, CD8^+^ T Cells, NK Cells, Macrophages, and DCs

Spleens from the tumor-bearing C57BL/6 mice or cryo-thermal treated mice were harvested and splenocytes were prepared using GentleMACS™ dissociator (Miltenyi Biotec, Bergisch Gladbach, Germany) and passed through a 40-μm nylon filter. CD4^+^ T cells were isolated by EasySep™ Mouse CD4 Positive Selection Kit II (StemCell Technologies, Vancouver, BC, Canada). CD8^+^ T cells were isolated by EasySep™ Mouse CD8^+^ T Cell Isolation Kit (StemCell Technologies, Vancouver, BC, Canada). Natural Killer (NK) cells were isolated by EasySep™ Mouse NK Cell Isolation Kit (StemCell Technologies, Vancouver, BC, Canada). CD68^+^ macrophages were isolated by EasySepTM PE positive selection kit (StemCell Technologies, Vancouver, BC, Canada) and CD68-PE (clone FA-11, Biolegend, San Diego, CA, USA). DCs were isolated by EasySep™ Mouse Pan-DC Enrichment Kit II (StemCell Technologies, Vancouver, BC, Canada). Cells were all isolated according to the manufacturer’s instructions. Cells with a purity of >90% were used for experiments.

### Adoptive T Cell Therapy

Splenic CD4^+^ T cells were separated on day 21 on 4T1 model after cryo-thermal therapy by magnetic-activated cell sorting (MACS) and were adoptive transferred into nude mice (1.5 million cells in 100 μL PBS per mice, i.v.). After 24 hours, the nude mice were inoculated with 50 thousand of 4T1 cells subcutaneously. The tumor volume was measured and calculated following formula: V (cm3) = π × L (major axis) × W (minor axis) × H (vertical axis)/6.

### Statistical Analysis

All data are presented as mean ± standard deviation (SD). Significance was determined using a two-sided Student’s T-test. GraphPad Prism 9.0 (La Jolla, CA) was used for all statistical analysis.

## Results

### Th1-Dominant CD4^+^ T Cells Mediated a Stronger Systematic Antitumor Immune Memory Than CD8^+^ T Cells After Local Cryo-Thermal Therapy

**Figure 1:**
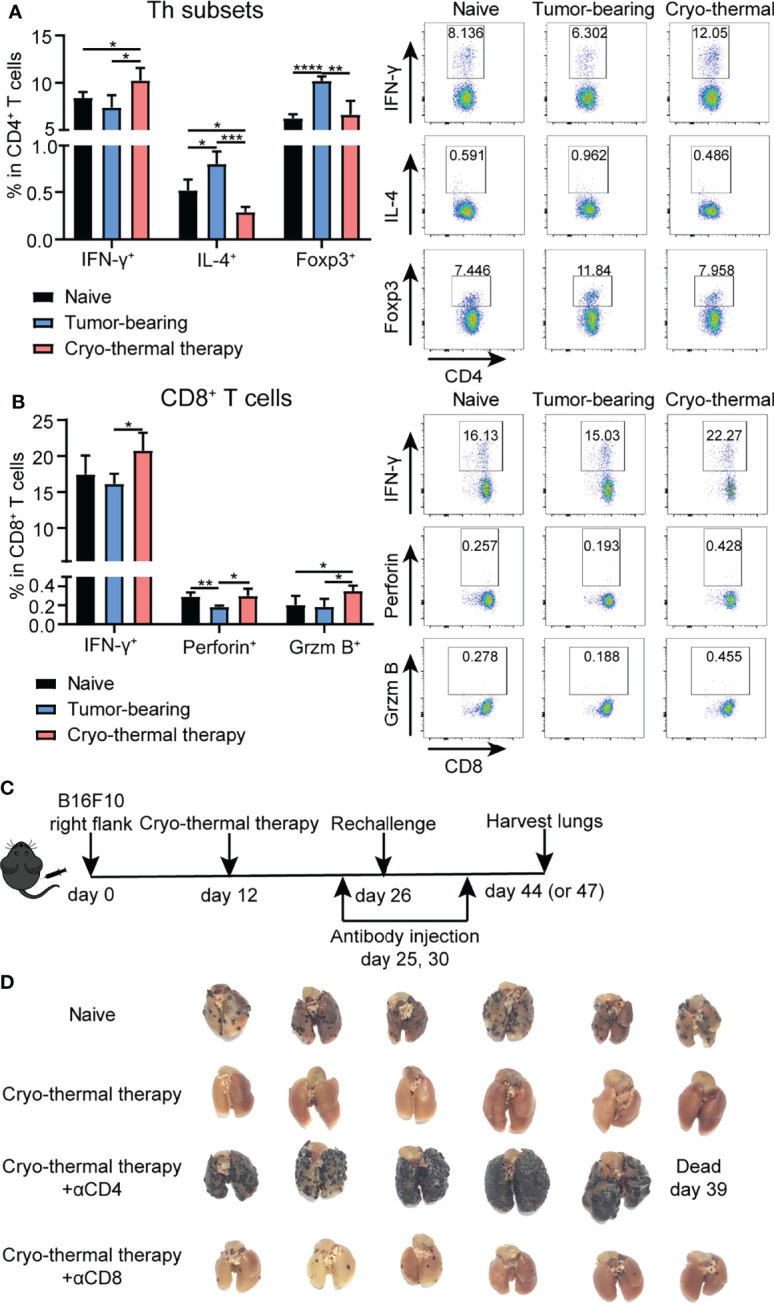
Phenotype of T cells after cryo-thermal therapy and tumor growth in rechallenge model depleted of T cells *in vivo*. Splenocytes of naïve, tumor bearing and cryo-thermal treated mice on day 14 after therapy were obtained to detect the phenotype of **(A)** CD4^+^ T cells; **(B)** CD8^+^ T cells by using flow cytometry. All data were shown as mean ± SD. *n*=4 for each group. *P < 0.05, **P < 0.01, ***P < 0.001, ****P < 0.0001. Data for graphs were calculated by using two-sided Student’s T-test. **(C)** Scheme of tumor rechallenge model. Approximately 5 × 10^5^ B16F10 cells were injected subcutaneously into the right flank of each mouse. Twelve days later, mice were treated with cryo-thermal therapy. Tumor rechallenge was conducted 14 days later, with 1 × 10^5^ B16F10 cells injected *via* tail vein. One day before and four days after tumor rechallenge, monoclonal antibodies were injected to deplete target cells or neutralize target cytokine. All mice were sacrificed on day 44 or 47 to detect tumor nodules in the lung and immune cells in the spleen. **(D)** Picture of tumor nodules in the lung after tumor rechallenge depleted of CD4^+^ T cells or CD8^+^ T cells. *n*=6 per group.

Previously, we demonstrated that CD4^+^ T cells were essential to mediate local antitumor immune memory, which led to better long-term survival rates upon local tumor rechallenge (14). Moreover, a further study revealed that neoantigen-specific CD4^+^ T cells are critical for the therapeutic efficacy of cryo-thermal therapy (13). However, how T cells mediate systematic antitumor immune memory after cryo-thermal therapy is unknown. Thus, the characteristics of splenic bulk CD4^+^ and CD8^+^ T cells on day 14 after the therapy were analyzed by flow cytometry (**Figure S1**). As depicted in **Figure 1A**, an increased percentage of CD4^+^ Th1 cells (interferon gamma, IFN-γ^+^) and a decreased percentage of Th2 cells (interleukin 4, IL-4^+^) and regulatory T (Treg) cells (Foxp3^+^) were observed. Meanwhile, the percentages of CD4^+^ cytotoxic T lymphocytes (CTLs) (Thpok^-^) and Th17 cells (IL-17^+^) were also increased, but the level of Th17 cells was much lower than that of CD4^+^ Th1 cells (**Figure S2A**). The percentage of T follicular helper (Tfh) cells (Bcl-6^+^) was significantly decreased (**Figure S2A**), which indicated that CD4^+^ T cells underwent a Th1-dominant response on day 14 after the therapy. The expression of the cytotoxic cytokines perforin and granzyme B in CD4^+^ T cells remained at a low level, thus suggesting that CD4^+^ T cells would not mediate their killing by the granzyme/perforin pathway (**Figure S2B**). On the other hand, the levels of IFN-γ, perforin and granzyme B in CD8^+^ T cells were significantly increased after cryo-thermal therapy (**Figure 1B**), which indicated that CD8^+^ T cells killed tumor cells *via* a perforin-dependent pathway. To further verify that cryo-thermal CD4^+^ or CD8^+^ T cells mediated systematic antitumor immune memory, mice were rechallenged with 1×10^5^ B16F10 tumor cells i.v. on day 14 after cryo-thermal therapy. Depletion of CD4^+^ or CD8^+^ T cells were performed using anti-CD4 or anti-CD8 monoclonal antibody injection i.p. one day before and four days after tumor rechallenge, respectively (**Figure 1C**). Efficacy of T cell depletion was verified by flow cytometry (**Figure S3A**). Lung tumor nodules were quantified 18 days later. First of all, as shown in **Figure 1D**, cryo-thermal treated mice could completely reject tumor rechallenge after intravenous tumor injection. Clearly, depletion of CD4^+^ T cells or CD8^+^ T cells abolished the antitumor effect induced by cryo-thermal therapy, leading to tumor growth in the lung. However, depletion of CD4^+^ T cells resulted in many more pulmonary tumor nodules in comparison with that with CD8^+^ T cells depletion, indicating more severely impaired the systematic antitumor protection (**Figure 1D**). These results showed that CD4^+^ Th1 cells were predominant after cryo-thermal therapy, CD4^+^ T cells played a critical role in systematic antitumor immune memory.

### Cryo-Thermal Th1-Dominant CD4^+^ T Cells Mediated the Differentiation and Function of Multiple Innate and Adaptive Immune Cells *In Vivo*

**Figure 2:**
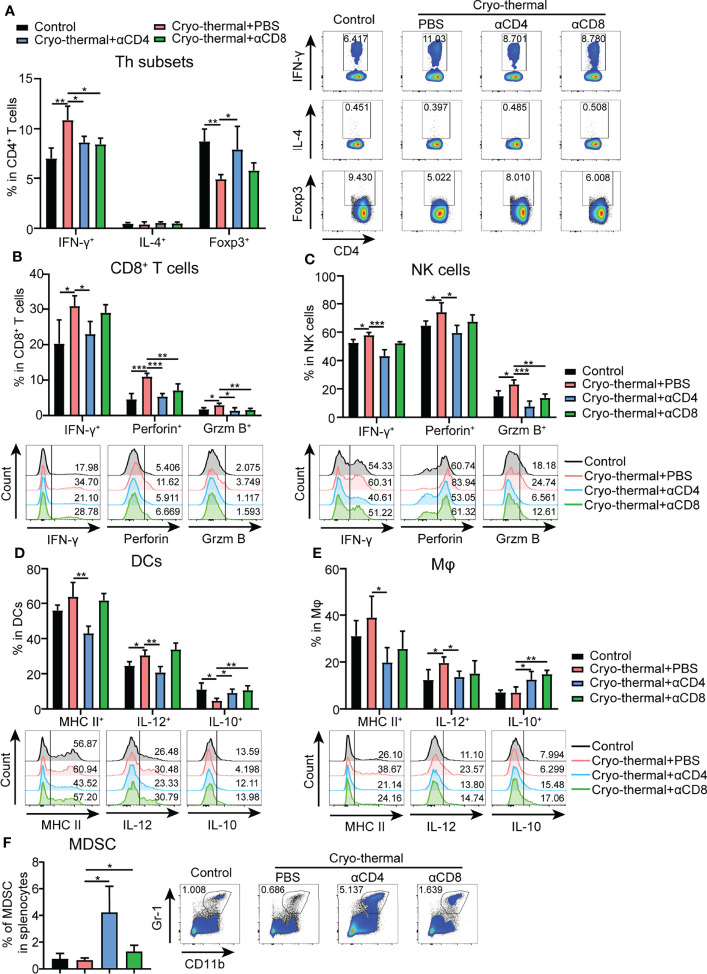
Phenotype of immune cells in tumor rechallenge model depleted of T cells after cryo-thermal therapy *in vivo*. Eighteen days after tumor rechallenge, the phenotype of **(A)** CD4^+^ T cells; **(B)** CD8^+^ T cells; **(C)** NK cells; **(D)** DCs; and **(E)** Mφs; and **(F)** frequency of MDSCs in the spleen. All data were shown as mean ± SD. n=4 for each group. *P < 0.05, **P < 0.01, ***P < 0.001, ****P < 0.0001. Data for graphs were calculated by using two-sided Student’s T-test.

The above results indicated that CD4^+^ T cells did not upregulate the expression of cytolytic molecules after cryo-thermal therapy, but CD4^+^ T cells could perform strong immunological memory against tumor rechallenge. How CD4^+^ T cells involved in the maintenance of antitumor immune memory was investigated as follows. To study the changes in immune cells in cryo-thermal treated mice after intravenous tumor rechallenge (as shown in **Figure 1D**), the spleens were harvested on day 18 after tumor rechallenge and the phenotypes of the immune cells analyzed. The total number of splenocytes were not altered significantly (**Figure S3B**). As shown in **Figure 2A**, the level of CD4^+^ T helper (Th) 1 cells in cryo-thermal treated mice was higher while the percentage of Tregs much lower than that in the control group (naive mice received tumor rechallenge). The other Th subsets, including Th2, Th17 and CTL subsets, showed no significant differences between cryo-thermal treated and controlled mice (**Figure 2A** and **Figure S3C**). These results indicated that CD4^+^ Th1 cells induced by cryo-thermal therapy were predominant over Tregs even after tumor rechallenge.

Upon depletion of CD4^+^ or CD8^+^ T cells prior to tumor rechallenge, as shown in **Figure 2A** and **Figure S3C**, the percentage of CD4^+^ Th1 cells was significantly decreased in cryo-thermal treated mice. On the other hand, the percentages of Tregs and Th17 cells were significantly increased by depletion of CD4^+^ T cells, but were not obviously changed by depletion of CD8^+^ T cells. However, the percentages of Th2, Tfh and CD4^+^ CTLs were not influenced by depletion of CD4^+^ T cells or CD8^+^ T cells. Also, the expression of perforin and granzyme B in CD4^+^ T cells induced by cryo-thermal therapy was not influenced by the depletion of CD4^+^ T or CD8^+^ T cells (**Figure S3C**). All these data suggested that the strong antitumor potential of cryo-thermal CD4^+^ T cells against tumor rechallenge could be attributed to CD4^+^ Th1-mediated antitumor immune memory. Although CD8^+^ T cells could modulate the differentiation of CD4^+^ T cells toward the Th1 phenotype, they could not affect Th1-dominant CD4^+^ T cell profile induced by cryo-thermal therapy.

In addition, after tumor rechallenge, the expression levels of IFN-γ, perforin, and granzyme B in CD8^+^ T cells in cryo-thermal treated mice were significantly increased, and they were significantly decreased with depletion of CD4^+^ T cells, but only perforin and granzyme B in CD8^+^ T cells were significantly decreased with depletion of CD8^+^ T cells (**Figure 2B**). Furthermore, the increased levels of IFN-γ, granzyme B and perforin in NK cells after cryo-thermal therapy were also significantly decreased with depletion of CD4^+^ T cells, while only the level of granzyme B in NK cells was significantly decreased with the depletion of CD8^+^ T cells (**Figure 2C**). All these results indicated that Th1-dominant CD4^+^ T cells induced by cryo-thermal therapy regulated the expression of IFN-γ in CD8^+^ T and NK cells and enhanced their cytotoxicity against tumor cells. CD8^+^ T cells slightly affected the cytotoxicity of NK cells.

After tumor rechallenge, the fraction of DCs and macrophages were not changed (**Figures S3D, E**). Moreover, the levels of major histocompatibility complex (MHC) class II and IL-12 in DCs and macrophages in cryo-thermal treated mice were significantly decreased with depletion of CD4^+^ T cells only, while the level of IL-10 in DCs and macrophages was significantly increased with depletion of CD4^+^ T cells or CD8^+^ T cells (**Figure 2D**), indicating that Th1-dominant CD4^+^ T cells induced by cryo-thermal therapy had a striking ability to promote DC maturation and M1 macrophage polarization in comparison to CD8^+^ T cells. After tumor rechallenge, the percentage of MDSCs was significantly increased with depletion of CD4^+^ T cells but not with depletion of CD8^+^ T cells, corresponding with the high tumor growth in lung shown in **Figure 1D** (**Figure 2F**). Collectively, these data suggested that after cryo-thermal therapy, Th1-dominant CD4^+^ T cells played a more extensive and principal role in regulating multiple innate and adaptive immune cell differentiation and maturation to mediate antitumor immune memory than CD8^+^ T cells.

### Cryo-Thermal Th1-Dominant CD4^+^ T Cells Enhanced the Mature Phenotype of Multiple Innate Immune Cells and the Cytotoxicity of NK Cells and CD8^+^ T Cells *In Vitro*

**Figure 3:**
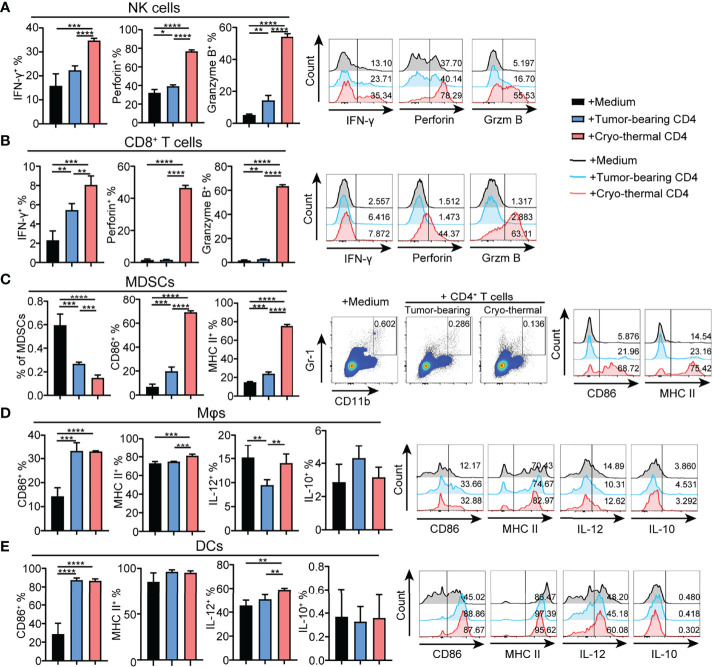
Phenotype of immune cells cocultured with CD4^+^ T cells *in vitro*. Cryo-thermal CD4^-^ cells were cocultured with tumor-bearing or cryo-thermal CD4^+^ T cells, and immune phenotype of **(A)** NK cells, **(B)** CD8^+^ T cells, **(C)** MDSCs, **(D)** Mφs and **(E)** DCs were analyzed. All data were shown as mean ± SD. *n*=4 for each group. *P < 0.05, **P < 0.01, ***P < 0.001, ****P < 0.0001. Data for graphs were calculated by using two-sided Student’s T-test.

To further identify the principal role of CD4^+^ T cells in regulating the differentiation and maturation of other immune cells, the phenotypes of other immune cells affected by CD4^+^ T cells from the tumor-bearing or cryo-thermal treated mice were studied *in vitro*. CD4^+^ T cells were isolated by MACS, and the remaining CD4^-^ cells were also collected. The phenotypes of NK cells, CD8^+^ T cells, DCs, macrophages and MDSCs in CD4^-^ splenocytes were further analyzed after coculturing with CD4^+^ T cells from the tumor-bearing or cryo-thermal treated mice, and comparisons made. The expression levels of IFN-γ, perforin and granzyme B in NK cells and CD8^+^ T cells were significantly upregulated (**Figures 3A, B**), and further cytotoxicity assay confirmed these results (**Figures S4A, B**). In addition, the percentage of MDSCs was significantly decreased after coculturing with cryo-thermal CD4^+^ T cells (**Figure 3C**). Moreover, the levels of MHC II and CD86 in MDSCs were significantly increased, suggesting that cryo-thermal CD4^+^ T cells could promote the maturation of MDSCs to reverse immunosuppression. Both tumor-bearing CD4^+^ T cells and cryo-thermal CD4^+^ T cells promoted the expression of CD86 in DCs and macrophages, but only cryo-thermal CD4^+^ T cells significantly upregulated the expression of MHC II in macrophages (**Figures 3D, E**). Cryo-thermal CD4^+^ T cells also induced high expression of IL-12 in DCs and maintained a high level of IL-12 in macrophages compared to tumor-bearing CD4^+^ T cells (**Figures 3D, E**). Overall, these *in vitro* studies verified that cryo-thermal Th1-dominant CD4^+^ T cells could enhance the cytotoxicity of NK cells and CD8^+^ T cells and promote the maturation of APCs, whereas they induced not only the destruction of MDSCs but also the maturation of MDSCs, leading to strong antitumor immune memory, which was similar to the *in vivo* results.

### Cryo-Thermal-Induced Th1-Dominant CD4^+^ T Cells Activated NK Cells and Regulated CD8^+^ T Cells *via* Cell-Cell Contact, and Promoted Maturation of Macrophages and DCs Partially *via* Cell-Cell Contact

**Figure 4:**
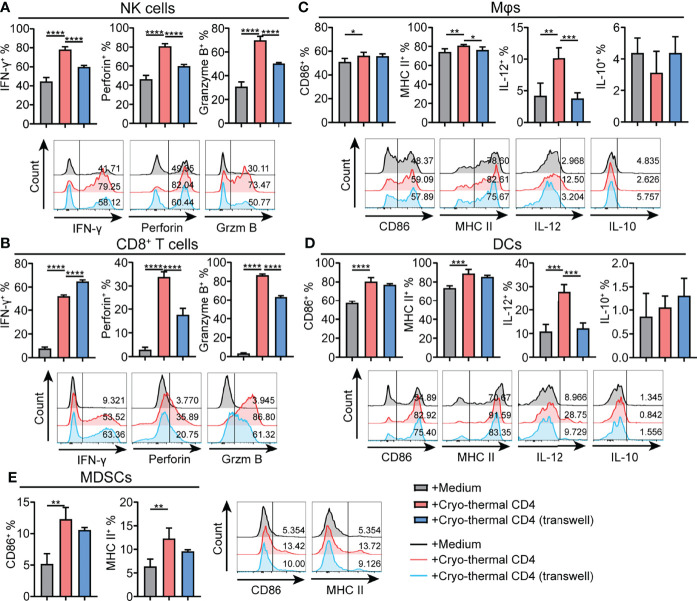
Phenotype of immune cells regulated by cryo-thermal CD4^+^ T cells is dependent on cell-cell contact *in vitro*. **(A-E)** MACS-isolated cryo-thermal CD4^+^ T cells were stimulated with anti-CD3 monoantibody, washed and then cocultured with cryo-thermal CD4^-^ splenocytes together or separated with a transwell plate for 24 hours. The phenotype of **(A)** NK cells, **(B)** CD8^+^ T cells, **(C)** Mφ, **(D)** DCs and **(E)** MDSCs were analyzed by flow cytometry. All data were shown as mean ± SD. *n*=4 for each group. *P < 0.05, **P < 0.01, ***P < 0.001, ****P < 0.0001. Data for graphs were calculated by using two-sided Student’s T-test.

As cryo-thermal Th1-dominant CD4^+^ T cells widely regulate other immune cells, it was further studied if such regulation had been *via* cell–cell contact. CD4^+^ T cells were isolated by using MACS, and the remaining CD4^-^ cells were also collected. Then, cryo-thermal CD4^-^ splenocytes were directly cocultured with cryo-thermal CD4^+^ T cells or separated with a transwell plate (0.4-μm pore size). As shown in **Figure 4A**, the expression of IFN-γ, perforin and granzyme B in NK cells was significantly decreased in transwell plates compared to normal plates, indicating that cryo-thermal CD4^+^ T cells activated NK cells *via* cell–cell contact. Meanwhile, the expression of perforin and granzyme B in CD8^+^ T cells was significantly decreased, but the expression of IFN-γ in CD8^+^ T cells was increased in the transwell plate compared to the normal plate (**Figure 4B**), which revealed that cryo-thermal CD4^+^ T cells promoted the cytotoxicity of CD8^+^ T cells but inhibited the production of IFN-γ *via* cell–cell contact. Moreover, the expression of MHC II and IL-12 in macrophages and the expression of IL-12 in DCs were significantly decreased in transwell plates compared to normal plates, suggesting that cryo-thermal CD4^+^ T cells could promote the maturation of APCs *via* cell–cell contact (**Figures 4C, D**). However, the levels of CD86 and MHC II in MDSCs were not significantly different in transwell plates and normal plates after coculture with cryo-thermal CD4^+^ T cells, revealing that cryo-thermal CD4^+^ T cells regulated the maturation of MDSCs through soluble factors. Overall, we discovered that cryo-thermal CD4^+^ T cells activated NK cells and regulated CD8^+^ T cells in a contact-dependent manner and promoted the maturation of macrophages and DCs partially *via* cell-cell contact but regulated the maturation of MDSCs through soluble factors.

### Cryo-Thermal CD4^+^ Th1 Cells Inhibited the Growth of Tumor Cells and the Differentiation of CD4^+^ T Cells Toward Other CD4^+^ Th Subsets Through CD4^+^ T Cell-Derived IFN-γ

**Figure 5:**
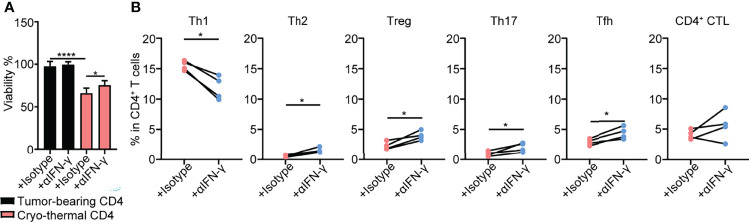
Tumor growth inhibition and Th subsets of CD4^+^ T cells *in vitro*. **(A)**Viability of tumor cells. Tumor cells were cocultured with tumor-bearing or cryo-thermal CD4^+^ T cells at 8:1 of E:T ratio in the presence of 10μg/mL isotype or anti-IFN-γ antibody. Twenty-four hours later, the viability of tumor cells was detected by CCK-8 Kit. **(B)** Percentage of Th subsets. Cryo-thermal CD4^+^ T cells were stimulated by 1μg/mL anti-CD3 antibody in the presence of 10μg/mL isotype or anti-IFN-γ antibody. Three days later, Th subsets were detected by flow cytometry. All data were shown as mean ± SD. *n*=4 for each group. *P < 0.05, **P < 0.01, ***P < 0.001, ****P < 0.0001. Data for graphs were calculated.

The above studies demonstrated that cryo-thermal Th1-dominant CD4^+^ T cells mediated the differentiation and function of multiple innate and adaptive immune cells. To further reveal how cryo-thermal CD4^+^ T cells affected tumor cells and maintained the Th1 subset, splenic CD4^+^ T cells in cryo-thermal treated mice and tumor-bearing mice were isolated. Because CD4^+^ Th1 cells are characterized by the secretion of IFN-γ, the isolated CD4^+^ T cells were incubated with B16F10 tumor cells *in vitro* in the presence of isotype or anti-IFN-γ antibody for 24 hours, and the viability of B16F10 cells was assessed by using CCK-8. As shown in **Figure 5A**, the tumor cell growth inhibition induced by cryo-thermal CD4^+^ T cells was much stronger than that induced by tumor-bearing CD4^+^ T cells, but *via* neutralization of IFN-γ, cryo-thermal CD4^+^ T cells promoted the growth of tumor cells. The cell cycle of tumor cells was also analyzed, and the result showed that CD4^+^ T cell derived IFN-γ could induce growth arrest in tumor cells (**Figures S5A, B**). These data revealed that cryo-thermal CD4^+^ T cells could directly inhibit the growth of tumor cells in an IFN-γ-dependent manner. Furthermore, we investigated how CD4^+^ T cells could maintain the CD4^+^ Th1 subset. The isolated splenic CD4^+^ T cells in cryo-thermal treated mice were stimulated with anti-CD3 antibody in the presence of isotype or anti-IFN-γ antibody. Three days later, CD4^+^ Th subsets were analyzed by flow cytometry. Interestingly, although the proportion of Th1 subset was significantly decreased, the levels of other CD4^+^ Th subsets, including Th2 cells, Tregs, Th17 cells and Tfh cells, were obviously increased *via* neutralization of IFN-γ. The level of CD4^+^ CTLs was not affected by the neutralization of IFN-γ (**Figure 5B**). These results indicated that cryo-thermal CD4^+^ Th1 cells inhibited the differentiation of CD4^+^ T cells toward other CD4^+^ Th subsets through CD4^+^ T-cell-derived IFN-γ.

### Cryo-Thermal CD4^+^ Th1 Cells Regulated the Antitumor Phenotype and Function of Other Immune Cells Through CD4^+^ T Cell-Derived IFN-γ

**Figure 6:**
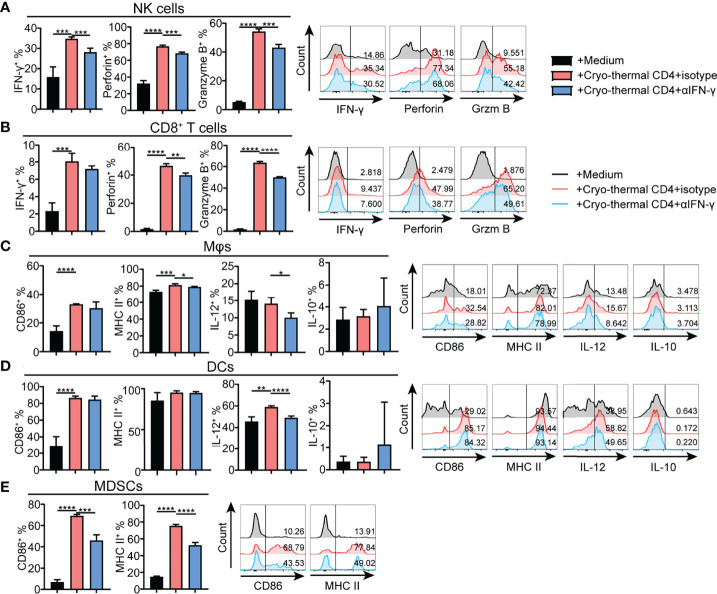
Phenotype of immune cells regulated by cryo-thermal CD4^+^ T cells derived IFN-γ *in vitro*. **(A-E)** MACS-isolated cryo-thermal CD4^+^ T cells were stimulated with anti-CD3 monoantibody, washed and then cocultured with cryo-thermal CD4^-^ splenocytes in the presence of 10 μg/mL isotype or anti-IFN-γ antibody for 24 hours. As a control, CD4^-^ splenocytes were cultured with 5 ng/mL recombination IFN-γ alone. The phenotype of **(A)** NK cells, **(B)** CD8^+^ T cells, **(C)** Mφ, **(D)** DCs and **(E)** MDSCs were analyzed by flow cytometry. All data were shown as mean ± SD. *n* =4 for each group. *P < 0.05, **P < 0.01, ***P < 0.001, ****P < 0.0001. Data for graphs were calculated by using two-sided Student’s T-test.

As cryo-thermal CD4^+^ Th1 cells secrete IFN-γ to maintain polarization of the CD4^+^ T-cell response toward Th1 dominance, the effect of IFN-γ secreted by cryo-thermal CD4^+^ Th1 cells on other immune cells was also studied *in vitro*. CD4^+^ T cells were isolated by MACS, and the remaining CD4^-^ cells were also collected. Cryo-thermal CD4^-^ splenocytes were cocultured with cryo-thermal CD4^+^ T cells in the presence of isotype or anti-IFN-γ antibody. Although the level of IFN-γ in CD8^+^ T cells was not changed with neutralization of IFN-γ, the increased level of IFN-γ in NK cells and the levels of perforin and granzyme B in NK and CD8^+^ T cells were impaired with neutralization of IFN-γ compared to the isotype control (**Figures 6A, B**). These results suggested that cryo-thermal CD4^+^ Th1 cells could activate NK cells and enhance the cytotoxicity of NK and CD8^+^ T cells through CD4^+^ T-cell-derived IFN-γ. The levels of IL-12 in DCs and macrophages and MHC II in macrophages were decreased with neutralization of IFN-γ compared to the isotype control (**Figures 6C, D**). These data revealed that cryo-thermal CD4^+^ Th1 cells could promote the functional maturation of APCs (with a high level of IL-12) in a CD4^+^ T-cell-derived IFN-γ-dependent manner. The levels of CD86 and MHC II in MDSCs were decreased with neutralization of IFN-γ compared to the isotype control (**Figure 6E**). These results indicated that cryo-thermal CD4^+^ Th1 cells could promote the maturation of DCs, macrophages and MDSCs through CD4^+^ T-cell-derived IFN-γ. As depicted in **Figures 4** and **6**, we concluded that cryo-thermal CD4^+^ T cells could be involved in the regulation of endogenous immune cells *via* cell–cell contact and CD4^+^ T-cell-derived IFN-γ. Although CD4^+^ T-cell-derived IFN-γ can regulate NK cells and CD8^+^ T cells (**Figures 6A, B**), cell–cell contact played a more important role in their cytotoxicity (**Figures 4A, B**). However, the maturation of MDSCs induced by cryo-thermal CD4^+^ T cells was mainly dependent on CD4^+^ T-cell-derived IFN-γ (**Figures 4E**, **6E**). Collectively, these data showed that cryo-thermal Th1-dominant CD4^+^ T cells could activate NK cells, enhance the cytotoxicity of NK and CD8^+^ T cells, promote the maturation of APCs through cell–cell contact and CD4^+^ T-cell-derived IFN-γ, and induce the maturation of MDSCs through CD4^+^ T-cell-derived IFN-γ.

### Adoptive Therapy Using Cryo-Thermal CD4^+^ T Cells Inhibited Tumor Growth *In Vivo*

**Figure 7:**
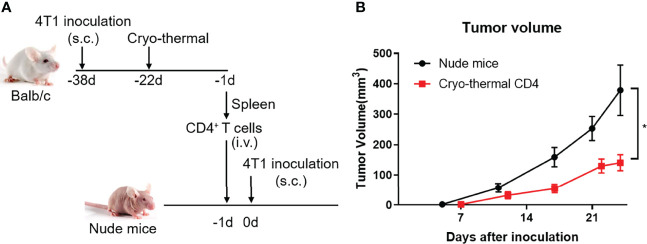
Adoptive therapy of cryo-thermal CD4^+^ T cells. **(A)** Scheme of study design. Splenic CD4^+^ T cells were separated on day 21 after cryo-thermal therapy by MACS and were adoptive transferred into nude mice (1.5 million cells in 100 μL PBS per mice, i.v.). After 24 hours, the nude mice were inoculated with 50 thousand of 4T1 cells subcutaneously. The tumor volume was measured and calculated following formula: V (cm^3^) = π × L (major axis) × W (minor axis) × H (vertical axis)/6. **(B)** Growth kinetics of a 4T1 breast cancer tumor model in nude mice as described. *p < 0.05. *n*=6 for each group.

As the role of CD4^+^ T cells after cryo-thermal therapy and their function in antitumor immunity are described above, we further determined the antitumor immunity of CD4^+^ T cells in T-cell-deficient hosts in 4T1 model, a model for the study of late-stage triple negative breast cancer (TNBC) (22). Cryo-thermal Th1-dominant CD4^+^ T cells from the 4T1 model 21 days after treatment were isolated by MACS and transferred into nude mice, and 1 day later, 4T1 cells were inoculated. Tumor size was measured every 5 days (**Figure 7A**). As shown in **Figure 7B**, cryo-thermal CD4^+^ T cells significantly decreased the growth of tumors *in vivo*. This result revealed that adoptively transferred cryo-thermal Th1-dominant CD4^+^ T cells, as effector cells, could mediate effective tumor rejection *in vivo*.

## Discussion

Cell-mediated immunity plays an important role in immune responses to prevent cancer. Activation of CD8^+^ cytotoxic T cells has long been regarded as a major antitumor mechanism of the immune system. It has become increasingly apparent that CD4^+^ T cells possess an extraordinary capacity to induce tumor rejection as principal effectors rather than as subsidiary helpers to cytolytic T cells. CD4^+^ T cells are much more effective in stimulating host immune responses to prevent tumor relapse than CD8^+^ T cells after immunotherapy (9–11, 23, 24). However, the role of CD4^+^ T cells in antitumor immune responses generated by various therapeutic strategies remains to be fully elucidated.

In this study, we demonstrated that after cryo-thermal therapy, CD4^+^ T cells orchestrated endogenous systematic antitumor immune memory and substantially reduced the accumulation of MDSCs and Tregs to reverse immunosuppression. CD4^+^ T cells can recognize peptides presented by professional APCs and differentiate into multiple subsets, such as Th1, Th2, Treg, Th17, CTL and Tfh cells (25–28). After cryo-thermal therapy, CD4^+^ Th1 cells dominated over other CD4^+^ subsets. Th1-polarized CD4^+^ T cells offer long-term protection against tumor rechallenge (29). CD4^+^ Th1 cells are characterized by the expression of the transcription Factor T-bet or signature cytokine IFN-γ, which are primarily responsible for activating and regulating the development and persistence of CTLs. In addition, Th1 cells activate APCs *via* costimulatory molecules (30). Our previous studies also showed that Th1-dominant CD4^+^ T cells mediate long-term antitumor immunity after cryo-thermal therapy (14). Tumor antigens and damage-associated molecular patterns (DAMPs) are released after local cryo-thermal therapy and induce strong neoantigen-specific Th1-dominant CD4^+^ T-cell antitumor immunity (13, 15). But the mechanism how CD4^+^ Th1 cells played the principal role in antitumor immune memory after cryo-thermal therapy were not understood. In this study, we comprehensively demonstrated that cryo-thermal induced Th1-dominant CD4^+^ T cells, as principal effector cells, inhibited tumor growth and exhibited multiple antitumor immunologic activities to enhance the cytotoxicity of CD8^+^ T and NK cells, promote the maturation of APCs and MDSCs, and decrease the levels of Tregs and MDSCs to maintain antitumor immune memory.

In this study, we suggested that the effect of Th1-dominant CD4^+^ T cells induced by cryo-thermal therapy could be mediated by Th1-cell-secreted soluble factors and cell-to-cell interactions. CD4^+^ T cells can regulate other immune cells through several contact-dependent pathways. CD40-CD40 L interactions are vital in the delivery of CD4^+^ T-cell help for many immune cells priming. CD4^+^ T cells constitutively express CD40 L and trigger DC maturation with the upregulation of MHC II and CD86 (31). The activation of macrophages by CD4^+^ T cells with the production of inflammatory cytokines and the generation of reactive nitrogen intermediates is dependent on CD40L (32). CD8^+^ T cells can receive CD4^+^ T cells to help directly through CD40, which is fundamental for CD8^+^ T-cell cytotoxic and memory generation (33, 34). In addition, CD4^+^ T cells can control the CD8^+^ T-cell response, resulting in a decrease in IFN-γ *via* a tumor necrosis factor-related apoptosis-inducing ligand (TRAIL)-mediated or Fas ligand (FasL)-dependent mechanism (35, 36), which would explain our *in vitro* results that cryo-thermal CD4^+^ T cells inhibited the production of IFN-γ *via* cell–cell contact (**Figure 4B**). Interestingly, depletion of CD8^+^ T cells showed little effect in antitumor events *in vivo*. We suggested that CD4^+^ T cells orchestrated comprehensive and diverse endogenous immune memory to inhibit tumor metastasis, including enhancing CD8^+^ CTL response, promoting NK cell activity and APC maturation. Thus, despite CD8^+^ T cells were depleted, CD4+ T cells could mobilize the other immune cells to inhibit the growth of tumor. We identified that cryo-thermal CD4^+^ T cells could inhibit the accumulation of MDSCs, and another study reported that activated T cells promoted MDSC apoptosis through the TRAIL–TRAILR pathway (8). We found that the interaction between CD4^+^ T cells and NK cells was extremely important to the activation and cytotoxicity of NK cells by CD4^+^ T cells; however, studies on CD4^+^ T-cell-to-NK-cell interactions have not been reported, and further study of the specific mechanism is needed.

Moreover, CD4^+^ T cells could modulate other immune cells through soluble factors. As the signature cytokine produced by CD4^+^ Th1 cells, IFN-γ can both directly mediate tumor rejection and recruit and activate innate and adaptive immune cells (37–41). Cryo-thermal CD4^+^ T cells directly inhibit the growth of tumor cells *via* CD4^+^ T-cell-derived IFN-γ, but the effect of IFN-γ alone seemed modest. It is possible that IFN-γ induced growth arrest in tumors synergistically with tumour necrosis factor-alpha (TNF-α) (3). In this study, we revealed that cell-derived IFN-γ maintained Th1-dominant CD4^+^ T cells induced by cryo-thermal therapy, and IFN-γ controls the expression of T-bet; at the same time, T-bet regulates IFN-γ production in an autocrine feedback loop, leading to the induction of the differentiation of the Th1 subset (42). Cryo-thermal CD4^+^ T cells enhanced the cytotoxicity of NK cells and CD8^+^ T cells and promoted the maturation of APCs and MDSCs *via* IFN-γ, which is in accordance with other studies (43). However, we noticed that neutralization of IFN-γ *in vitro* only partially abolished the effect of cryo-thermal CD4^+^ T cells on other immune cells, indicating that except through CD4^+^ T-cell-derived IFN-γ, Th1-dominant CD4^+^ T cells would perform antitumor immunity through other factors. IL-2 secreted by CD4^+^ Th1 cells also helps maintain the activation and cytotoxicity of CD8^+^ T cells and NK cells (44–47). Moreover, activated CD4^+^ Th1 cells can promote the recruitment and infiltration of CD8^+^ T cells, macrophages and NK cells through the chemokines C-X-C motif chemokine ligand (CXCL) 10 and CXCL9 (46, 48–51). Importantly, cryo-thermal CD4^+^ T cells could promote the maturation of MDSCs with upregulation of MHC II and CD86 *via* IFN-γ. Although some studies have shown that IFN-γ inhibits the immunosuppressive function of tumor-induced MDSCs (52), cryo-thermal CD4^+^ T cells regulating the maturation of MDSCs should be addressed. Additionally, although the level of CD4^+^ Th1 cells was decreased after depletion of CD8^+^ T cells when the cryo-thermal treated mice received tumor rechallenge, the level of Treg and Th17 were not influenced, which suggested that CD8^+^ T cells did not affect Th1 dominance in Th subsets after cryo-thermal therapy. CD8^+^ T cells modulate CD4^+^ T-cell immune responses *in vivo*, thus promoting their early activation and Tfh differentiation (53). However, the role of CD8^+^ T cells in regulating CD4^+^ Th1 differentiation should be further studied. Thus, the specific molecular mechanism of how cryo-thermal therapy induced Th1-dominant CD4+ T cells would be further studied in near future.

The functional status of CD4^+^ T cells is a critical determinant of antitumor immunity. The stimulation of the Th1 response in cancer immunotherapy is becoming increasingly important because the Th1 response can shift the direction of adaptive immune responses toward protective immunity. Polyfunctional CD4^+^ T cells with the ability to produce multiple Th1-type cytokines exhibit many desirable features for cancer immunotherapy. CD4^+^ Th1 cells exert powerful antitumor immune effects against numerous types of cancers (54–57). However, how to efficiently activate CD4^+^ T cells with multiple antitumor mechanisms *in vivo* has not been discovered, especially how to induce the differentiation of the Th1 dominant subset, which has not been defined (58, 59). Our study showed that polyfunctional Th1-dominant CD4^+^ T cells were induced after cryo-thermal therapy to effectively control distant tumor metastasis. The Th1 response induces epitope spreading to prevent tumor relapse due to antigen escape (60).This study highlighted that cryo-thermal Th1-dominant CD4^+^ T cells improved CTL generation as well as APC maturation to augment antitumor responses in the replacement of typical maturation reagents *in vitro*, and adoptively transferred cryo-thermal CD4^+^ T cells significantly decreased the growth of tumors *in vivo*, which suggest that cryo-thermal therapy could be further developed as a thermo-immunotherapy for clinical application. Current study is limited in B16F10 model and additional data from other qualifying murine models would be required to confirm the role of CD4^+^ T cells in antitumor immune memory.

In summary, Th1-dominant CD4^+^ T cells induced by cryo-thermal therapy orchestrated comprehensive and diverse endogenous antitumor immune memory to inhibit tumor metastasis. Th1-dominant CD4^+^ T cells induced by cryo-thermal therapy inhibited the tumor growth, enhanced the cytotoxicity of CD8^+^ T and NK cells, promoted the maturation of APCs and MDSCs, and decreased the levels of Tregs and MDSCs to maintain antitumor immune memory.

## Data Availability Statement

The original contributions presented in the study are included in the article/**Supplementary Material**. Further inquiries can be directed to the corresponding authors.

## Ethics Statement

All animal experiments were approved by the Animal Welfare Committee of Shanghai Jiao Tong University, and experimental methods were performed in accordance with the guidelines of Shanghai Jiao Tong University Animal Care (approved by Shanghai Jiao Tong University Scientific Ethics Committee).

## Author Contributions

PP, YL, JW, and SW performed experiments. PL coordinated the project. PL and PP designed experiments. Manuscript was written by PP and revised by PL, and LX. All authors reviewed the manuscript.

## Funding

This research was funded by the National Key Research and Development Program of China (Grant No. 2020YFA0909003), National Natural Science Foundation of China (Grant No. 82072085), and the Shanghai Science and Technology Commission of Shanghai Municipality (Grant No. 19DZ2280300).

## Conflict of Interest

The authors declare that the research was conducted in the absence of any commercial or financial relationships that could be construed as a potential conflict of interest.

## Publisher’s Note

All claims expressed in this article are solely those of the authors and do not necessarily represent those of their affiliated organizations, or those of the publisher, the editors and the reviewers. Any product that may be evaluated in this article, or claim that may be made by its manufacturer, is not guaranteed or endorsed by the publisher.

## Abbreviations

APC, antigen presenting cells; BFA, Brefeldin A; CAR, chimeric antigen receptor; CCK-8, Cell Counting Kit-8; CD40L, CD40 ligand; CTL, cytotoxic T lymphocyte; CXCL, C-X-C motif chemokine ligand; DAMP, damage-associated molecular pattern; DC, dendritic cell; DMEM, Dulbecco’s Modified Eagle’s Medium; FasL, Fas ligand; FBS, fetal bovine serum; IFN-γ, interferon gamma; IL, interleukin; i.p., intraperitoneal injection; mAb, monoclonal antibody; MACS, magnetic-activated cell sorting; MDSC, myeloid-derived suppressor cells; MHC, major histocompatibility complex; NK cell, Natural Killer cell; RF, Radiofrequency; TCR, T cell receptor; Tfh, T follicular helper; Th, T helper; TNF-α, tumour necrosis factor-alpha; Treg, Regulatory T cell; TRAIL, tumor necrosis factor-related apoptosis-inducing ligand
