# Supplementary material for: Th1-Dominant CD4+ T Cells Orchestrate Endogenous Systematic Antitumor Immune Memory After Cryo-Thermal Therapy
Source: Front Immunol. 2022 Jul 8;13:944115. doi: 10.3389/fimmu.2022.944115 (PMC9304863; doi:10.3389/fimmu.2022.944115)
Supplement: Supplementary file 1 [file DataSheet_1.pdf]

Supplementary Material

1 Key Resource Table

REAGENT or RESOURCE	SOURCE	IDENTIFIER	
**Antibodies**			**Dilution**
FITC anti-mouse CD3ε Antibody (clone: 145-2C11)	BioLegend	Cat# 100306, RRID:AB_312671	2:100
PerCP/Cyanine5.5 anti-mouse CD3ε Antibody (clone: 145-2C11)	BioLegend	Cat# 100328, RRID:AB_893318	1.25:100
APC/Cyanine7 anti-mouse CD4 antibody (clone: RM4-5)	BioLegend	Cat# 100526, RRID:AB_312727	5:100
APC/Cyanine7 anti-mouse CD8a antibody (clone: 53-6.7)	BioLegend	Cat# 100714, RRID:AB_312753	5:100
PerCP/Cyanine5.5 anti-mouse CD8a antibody (clone: 53-6.7)	BioLegend	Cat# 100734, RRID:AB_2075238	5:100
Pacific Blue™ anti-mouse CD8a Antibody (clone: 53-6.7)	BioLegend	Cat# 100725, RRID:AB_493425	0.5:100
FITC anti-mouse NK-1.1 antibody (clone: PK136)	BioLegend	Cat# 108706, RRID:AB_313393	1:100
PE/Cyanine7 anti-mouse CD25 Antibody (clone: 3C7)	BioLegend	Cat# 101916, RRID:AB_2616762	2.5:100
Alexa Fluor® 647 anti-human/mouse Granzyme B Antibody (clone: GB11)	BioLegend	Cat# 515406, RRID:AB_2566333	5:100
PE anti-mouse Perforin Antibody (clone: S16009B)	BioLegend	Cat# 154406, RRID:AB_2721641	2.5:100
Brilliant Violet 421™ anti-mouse CD69 antibody (clone: H1.2F3)	BioLegend	Cat# 104528, RRID:AB_2562328	5:100
PE anti-mouse FOXP3 antibody (clone: MF-14)	BioLegend	Cat# 126404, RRID:AB_1089117	5:100
PE/Cy7 anti-mouse CD279 (PD-1) antibody (clone: 29F.1A12)	BioLegend	Cat# 135216, RRID:AB_10689635	1.25:100
Brilliant Violet 421™ anti-mouse IL-4 Antibody (clone: 11B11)	BioLegend	Cat# 504120, RRID:AB_2562102	5:100
PE anti-mouse IL-17A Antibody (clone: TC11-18H10.1)	BioLegend	Cat# 506904, RRID:AB_315464	1.25:100
APC anti-mouse IL-17A antibody (clone: TC11-18H10.1)	BioLegend	Cat# 506916, RRID:AB_536018	1.25:100
Pacific Blue™ anti-mouse/human CD11b Antibody (clone: M1/70)	BioLegend	Cat# 101224, RRID:AB_755986	2:100
FITC anti-mouse/human CD11b antibody (clone: M1/70)	BioLegend	Cat# 101206, RRID:AB_312789	0.5:100
PE anti-mouse Ly-6G/Ly-6C (Gr-1) Antibody (clone: RB6-8C5)	BioLegend	Cat# 108407, RRID:AB_10866471	1.25:100
PE anti-mouse CD11c Antibody (clone: N418)	BioLegend	Cat# 117308, RRID:AB_313777	0.5:100
FITC anti-mouse CD11c Antibody (clone: N418)	BioLegend	Cat# 117306, RRID:AB_313775	1.25:100
APC anti-mouse F4/80 antibody (clone: BM8)	BioLegend	Cat# 123116, RRID:AB_893481	1.25:100
PE/Cyanine7 anti-mouse Ly-6G Antibody (clone: 1A8)	BioLegend	Cat# 127617, RRID:AB_1877262	1.25:100

APC anti-mouse Ly-6G Antibody (clone: 1A8)	BioLegend	Cat# 127614, RRID:AB_2227348	0.5:100
FITC anti-mouse Ly-6C Antibody (clone: HK1.4)	BioLegend	Cat# 128005, RRID:AB_1186134	0.5:100
PerCP/Cyanine5.5 anti-mouse Ly-6C Antibody (clone: HK1.4)	BioLegend	Cat# 128012, RRID:AB_1659241	1.25:100
PerCP/Cyanine5.5 anti-mouse I-A/I-E antibody (clone: M5/114.15.2)	BioLegend	Cat# 107626, RRID:AB_2191071	0.3:100
APC/Cyanine7 anti-mouse CD86 Antibody (clone: GL-1)	BioLegend	Cat# 105030, RRID:AB_2244452	1.25:100
Brilliant Violet 421™ anti-mouse IL-10 antibody (clone: JES5-16E3)	BioLegend	Cat# 505022, RRID:AB_2563240	2.5:100
PE/Cy7 anti-mouse IL-12/IL-23 p40 antibody (clone: C15.6)	BioLegend	Cat# 505210, RRID:AB_2565645	2.5:100
PE/Dazzle™ 594 anti-T-bet Antibody (clone: 4B10)	BioLegend	Cat# 644828, RRID:AB_2565677	5:100
Alexa Fluor® 488 anti-GATA3 Antibody (clone: 16E10A23)	BioLegend	Cat# 653808, RRID:AB_2563215	5:100
IL-21 Monoclonal Antibody (clone: FFA21)	Thermo Fisher Scientific	Cat# 17-7211-82, RRID:AB_2016709	1.5:100
BV421 Mouse Anti-Bcl-6 (clone: K112-91)	BD Biosciences	Cat# 563363, RRID:AB_2738159	5:100
Alexa Fluor® 647 Rat Anti-Mouse Zbtb7b (ThPok) (clone: T43-94)	BD Biosciences	Cat# 565500, RRID:AB_2739268	5:100
APC Rat Anti-Mouse IL-2 (clone: JES6-5H4)	BD Biosciences	Cat# 554429, RRID:AB_398555	5:100
PE-CF594 Rat Anti-Mouse IFN-γ (clone: XMG1.2)	BD Biosciences	Cat# 562303, RRID:AB_11153140	1.25:100
PE anti-mouse CD68 Antibody (clone: FA-11)	BioLegend	Cat# 137014, RRID:AB_10612937	N/A
Ultra-LEAF™ Purified anti-mouse CD3ε Antibody (clone: 145-2C11)	BioLegend	Cat# 100340, RRID:AB_11149115	N/A
Ultra-LEAF™ Purified anti-mouse CD4 Antibody (clone: GK1.5)	BioLegend	Cat# 100457, RRID:AB_2810318	N/A
Ultra-LEAF™ Purified anti-mouse CD8a Antibody (clone: 53-6.7)	BioLegend	Cat# 100763, RRID:AB_2810323	N/A
Ultra-LEAF™ Purified anti-mouse IFN-γ Antibody (clone: XMG1.2)	BioLegend	Cat# 505847, RRID:AB_2616675	N/A
Ultra-LEAF™ Purified Rat IgG1, κ Isotype Ctrl Antibody	BioLegend	Cat# 400457	N/A
**Chemicals, Peptides, and Recombinant Proteins**			
Cell Activation Cocktail (with Brefeldin A)	BioLegend	Cat# 423304	
Recombinant Mouse IFN-γ (carrier-free)	BioLegend	Cat# 575304	
Dimethyl sulfoxide ACS	Avantor®	Cat# 97061-250	
Calcein-AM	Sigma-Aldrich	Cat# 17783	
Cell Counting Kit-8	Beyotime Biotechnology	Cat# C0038	
**Critical Commercial Assays**			
Fixation Buffer	BioLegend	Cat# 420801	
Intracellular Staining Permeabilization Wash Buffer (10X)	BioLegend	Cat# 421002	
True-Nuclear™ Transcription Factor Buffer Set	BioLegend	Cat# 424401	
Zombie Aqua™ Fixable Viability Kit	BioLegend	Cat# 423102	

EasySep™ Mouse CD4 Positive Selection Kit II	STEMCELL	Cat# 18952
EasySep™ Mouse CD8+ T Cell Isolation Kit	STEMCELL	Cat# 19853
EasySep™ Mouse NK Cell Isolation Kit	STEMCELL	Cat# 19855
EasySep™ Mouse Pan-DC Enrichment Kit	STEMCELL	Cat# 19763
EasySep™ Release Mouse PE Positive Selection Kit	STEMCELL	Cat# 17656
EasySep™ Mouse APC Positive Selection Kit II	STEMCELL	Cat# 17667
**Experimental Models: Organisms/Strains**		
Mouse: C57BL/6JSlac	Shanghai SLAC Laboratory Animal Co.,Ltd	N/A
Mouse: BALB/cSlac	Shanghai SLAC Laboratory Animal Co.,Ltd	N/A
Tumor cell line: 4T1	Shanghai First People's Hospital	N/A
Tumor cell line: B16F10	Donated by Pro. Weihai Ying Lab	N/A
Fetal Bovine Serum	ScienCell Research Laboratories	Cat# 0500
HyClone Penicillin-Streptomycin 100X solution	Cytiva life sciences	Cat# SV30010
DMEM/High glucose with L-glutamine, sodium pyruvate	Cytiva life sciences	Cat# SH30243.FS
RPMI 1640 medium with HEPES, L-glutamine	Cytiva life sciences	Cat# SH30255.FS
**Software and Algorithms**		
GraphPad Prism 8.0	GraphPad	RRID:SCR_002798 https://www.graphpad.com/scientific-software/prism/
FlowJo v 10	BD Biosciences	RRID:SCR_008520 https://www.flowjo.com/solutions/flowjo
ADOBE ILLUSTRATOR	Adobe	RRID:SCR_010279 https://www.adobe.com/cn/
FACSDiva	BD Biosciences	RRID:SCR_001456 https://www.bdbiosciences.com/en-us/instruments/research-instruments/research-software/flow-cytometry-acquisition/facsdiva-software

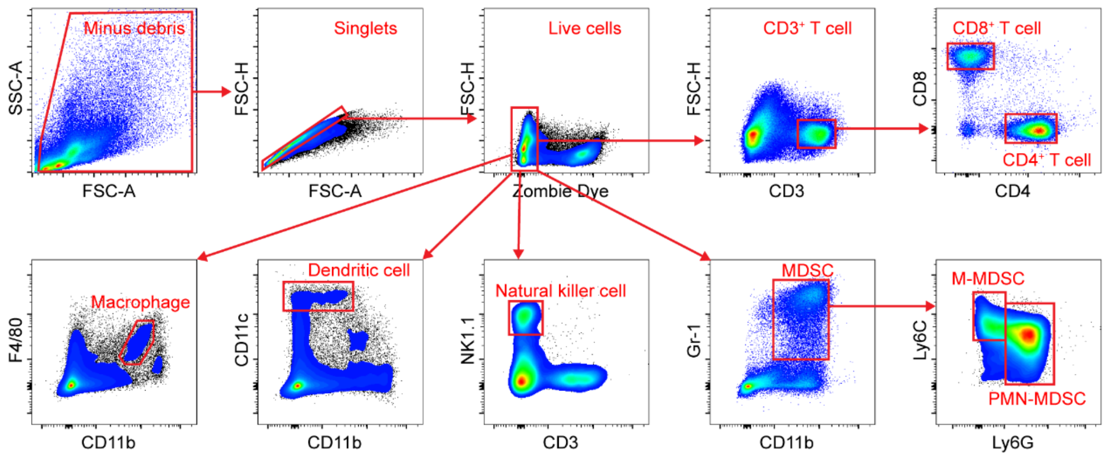

Supplementary Figure 1. Gating strategy of flow cytometry. Splenocytes of mice were obtained to prepare single-cell suspension and stained with fluorescence conjugated antibodies.

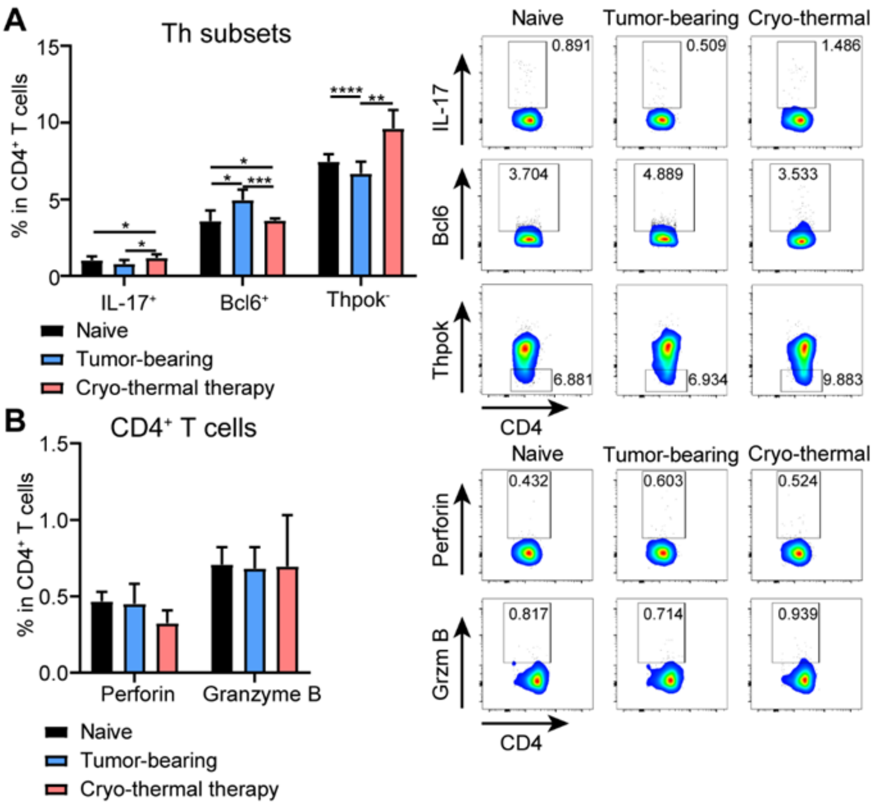

Supplementary Figure 2. Phenotype of $CD4^+$ T cells after cryo-thermal therapy. Splenocytes of naïve, tumour bearing and cryo-thermal treated mice on day 14 after therapy were obtained to detect the phenotype of T cells by using flow cytometry. (**A**) Frequency of IL-17^+, $Bcl6^+$ and $Thpok^-$ cells in $CD4^+$ T cells. (**B**) Frequency of $perforin^+$ and granzyme B^+ cells in $CD4^+$ T cells. All data were shown as mean ± SD. *n*=4 for each group. *P<0.05, **P<0.01, ***P<0.001, ****P<0.0001. Data for graphs were calculated by using two-sided Student's T-test.

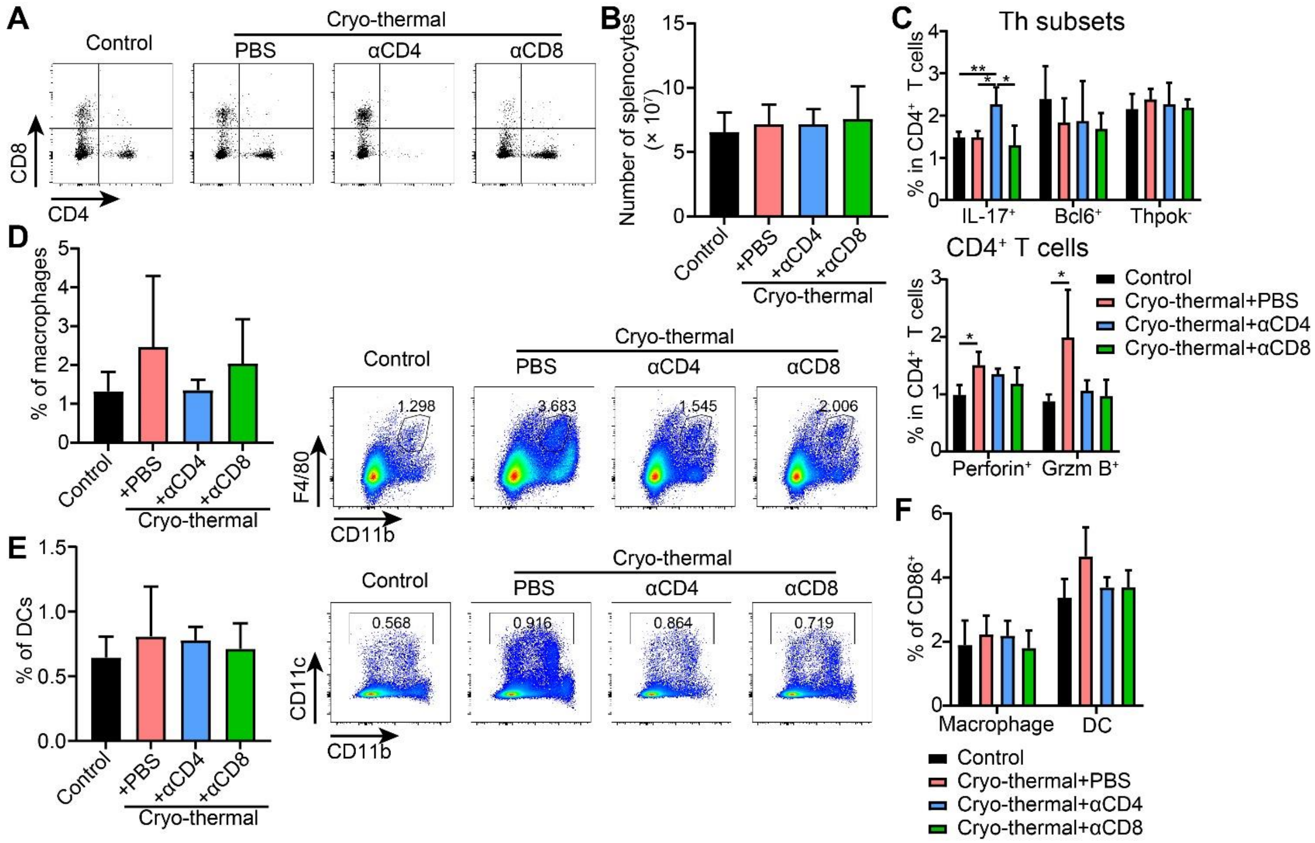

Supplementary Figure 3. Phenotype of immune cells in tumour rechallenge model depleted of T cells after cryo-thermal therapy *in vivo*. (**A**) T cell depletion verification. Eighteen days after tumour rechallenge, the number of total splenocytes (**B**), the phenotype of $CD4^+$ Th subsets (**C**), percentage of Mφs (**D**), DC (**E**) and phenotype of DCs and Mφs (**F**) in the spleen. All data were shown as mean ± SD. *n*=4 for each group. *P<0.05, **P<0.01, ***P<0.001, ****P<0.0001. Data for graphs were calculated using two-sided Student's T-test.

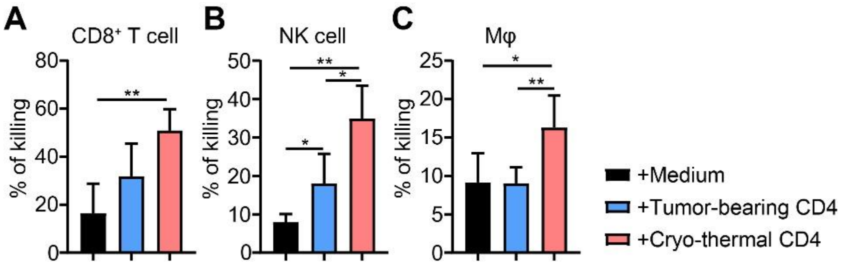

Supplementary Figure 4. Cytotoxicity of immune cells cocultured with CD4^{+} T cells *in vitro*. For the tumour cell killing assay, tumour cells were labelled with 1 μM calcein-AM and cocultured with effector cells for 4 hours. **(A-C**) Percentage of tumour cells killed by combination of CD8^{+} T cells (**A**), NK cells (**B**) or macrophages (**C**) with tumour-bearing or cryo-thermal CD4^{+} cells or alone. All data were shown as mean ± SD. *n*=4 for each group. *P<0.05, **P<0.01, ***P<0.001, ****P<0.0001. Data for graphs were calculated by using two-sided Student's T-test.

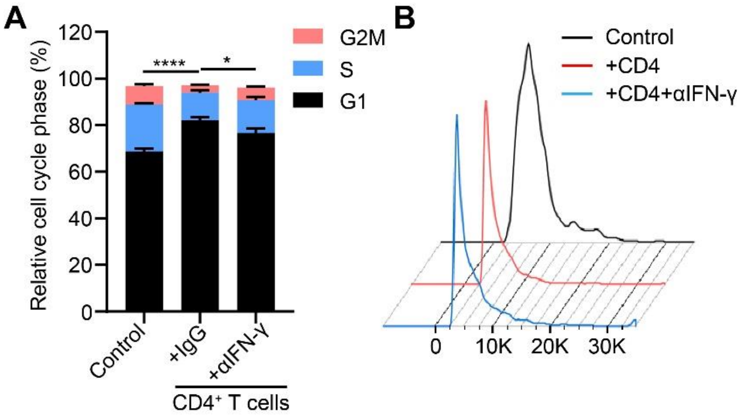

Supplementary Figure 5. Tumor cell cycle arrest by CD4+ T cells *in vitro*. B16F10 tumor cells were cocultured with cryo-thermal CD4+ T cells for 24 hours and were collected to analyse cell cycle by flow cytometry. (**A**) Proportion of tumor cells in G1, S and G2/M phase. (**B**) Representative flow cytometry graph of (**A**). All data were shown as mean ± SD. *n*=4 for each group. *P<0.05, **P<0.01, ***P<0.001, ****P<0.0001. Data for graphs were calculated by using two-sided Student's T-test.
